# Modeling of Nitrous Oxide Production from Nitritation Reactors Treating Real Anaerobic Digestion Liquor

**DOI:** 10.1038/srep25336

**Published:** 2016-04-29

**Authors:** Qilin Wang, Bing-Jie Ni, Romain Lemaire, Xiaodi Hao, Zhiguo Yuan

**Affiliations:** 1 Advanced Water Management Centre (AWMC), The University of Queensland, QLD 4072, Australia; 2 Veolia Technical and Performance Department, St-Maurice, France; 3 Key Laboratory of Urban Stormwater System and Water Environment/R&D Centre for Sustainable Wastewater Treatment (Beijing University of Civil Engineering and Architecture), Ministry of Education, Beijing 100044, P.R. China

## Abstract

In this work, a mathematical model including both ammonium oxidizing bacteria (AOB) and heterotrophic bacteria (HB) is constructed to predict N_2_O production from the nitritation systems receiving the real anaerobic digestion liquor. This is for the first time that N_2_O production from such systems was modeled considering both AOB and HB. The model was calibrated and validated using experimental data from both lab- and pilot-scale nitritation reactors. The model predictions matched the dynamic N_2_O, ammonium, nitrite and chemical oxygen demand data well, supporting the capability of the model. Modeling results indicated that HB are the dominant contributor to N_2_O production in the above systems with the dissolved oxygen (DO) concentration of 0.5–1.0 mg O_2_/L, accounting for approximately 75% of N_2_O production. The modeling results also suggested that the contribution of HB to N_2_O production decreased with the increasing DO concentrations, from 75% at DO = 0.5 mg O_2_/L to 25% at DO = 7.0 mg O_2_/L, with a corresponding increase of the AOB contribution (from 25% to 75%). Similar to HB, the total N_2_O production rate also decreased dramatically from 0.65 to 0.25 mg N/L/h when DO concentration increased from 0.5 to 7.0 mg O_2_/L.

Nitrous oxide (N_2_O) is not only a potent greenhouse gas, with a global warming potential of approximately 265 times stronger than carbon dioxide^1^, but also a compound destructing the stratospheric ozone layer^2^. N_2_O can be produced in the wastewater treatment systems through both nitrification and denitrification^3^,^4^,^5^,^6^,^7^. During nitrification, ammonium (NH_4_^+^) is first being oxidized to nitrite (NO_2_^−^) by ammonium-oxidizing bacteria (AOB). Afterwards, nitrite is further oxidized to nitrate (NO_3_^−^) by nitrite-oxidizing bacteria (NOB). N_2_O is not an obligatory intermediate of nitrification, but it can be generated by AOB via two primary pathways: i) N_2_O as the end product of AOB denitrification, called the nitrifier denitrification pathway, and ii) N_2_O as the by-product of incomplete oxidation of hydroxylamine (NH_2_OH), known as the NH_2_OH pathway^7^,^8^,^9^. By contrast, N_2_O is an obligatory intermediate of heterotrophic denitrification, which is composed of reductive reactions transforming NO_3_^−^ to NO_2_^−^, nitric oxide (NO), N_2_O and finally to nitrogen gas (N_2_). These reactions are carried out by heterotrophic bacteria (HB). N_2_O can accumulate when the N_2_O production is faster than the N_2_O reduction^6^,^7^,^8^,^10^.

It has become a common practice to remove nitrogen from the anaerobic digestion liquor in a side-stream process in wastewater treatment plants (WWTPs)^11^,^12^. The anaerobic digestion liquor has a high ammonium concentration of 300–1500 mg N/L and a low ratio of chemical oxygen demand to nitrogen (COD/N) for the conventional nitrification and denitrification process. One treatment option is partial nitritation (NH_4_^+^→NO_2_^−^) followed by the anammox process^12^,^13^. The partial nitritation process oxidizes approximately 50% of the ammonium to nitrite. This generates a mixture of nitrite and ammonium with a molar ratio of approximately 1:1, which is suitable for the subsequent anammox process. The other treatment option is nitritation followed by denitritation with the additional addition of an external carbon source such as methanol^14^.

N_2_O emissions from the nitritation systems receiving the anaerobic digestion liquor have been extensively reported. However, the results show a huge variation. For instance, the N_2_O emission factors (mg N_2_O-N emitted per mg NH_4_^+^-N oxidized) were determined to be from 0.7 to 19.3% of the NH_4_^+^-N oxidized^3^,^12^,^14^,^15^,^16^,^17^,^18^,^19^. Mathematical modeling is an appropriate method for estimating site-specific N_2_O emissions and is of great importance towards a full understanding of N_2_O production mechanisms from the nitritation reactors treating anaerobic digestion liquor. However, little effort has been dedicated to modeling N_2_O production from such systems, which are characterized by high nitrite accumulation and are significantly different from the main-stream wastewater treatment systems. Although Ni *et al.*^9^ modeled N_2_O production from such systems using an electron carrier (EC)-based mathematical model, the modeled nitritation system received organics-free synthetic digestion liquor. Therefore, the model did not include N_2_O production by heterotrophic bacteria (HB), which were recently experimentally demonstrated to play an important role in N_2_O production from such systems^3^. Pan *et al.*^20^ recently developed an EC-based denitrification model to model N_2_O production by HB in a methanol and nitrate-fed denitrifying culture. This model has been proven to be able to better predict N_2_O production than the commonly used four-step denitrification model^21^,^22^. However, this model has never been applied to model N_2_O production from the nitritation reactors treating anaerobic digestion liquor.

In this work, we aim to develop and calibrate a mathematical model to predict N_2_O production from the nitritation reactors receiving the real anaerobic digestion liquor. This model integrated the EC-based N_2_O production model for AOB with that for HB for the first time. The model was calibrated and validated by comparing the simulation results with the measured data from two different nitritation reactors (4 L and 500 L, respectively). The contributions of AOB and HB to N_2_O production as well as N_2_O production rates of both AOB and HB at various dissolved oxygen (DO) levels from such systems were also evaluated.

## Results

### Model calibration

**Figure 1:**
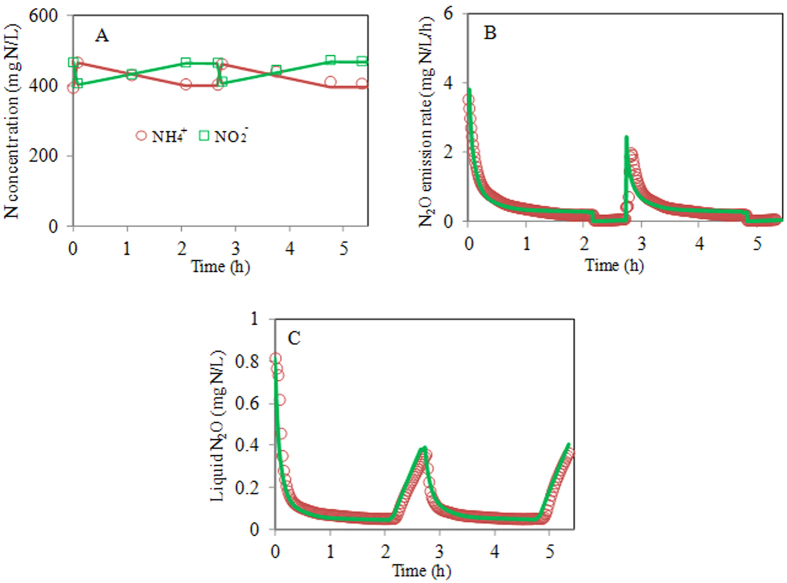
Model calibration results using the experimental data from Nitritation reactor I at DO = 0.5 mg O_2_/L (measured data: symbols; model predictions: lines): (**A**) NH_4_^+^ and NO_2_^−^ data; (**B**) Volumetric N_2_O emission rate; (**C**) Liquid phase N_2_O concentration.

**Figure 2:**
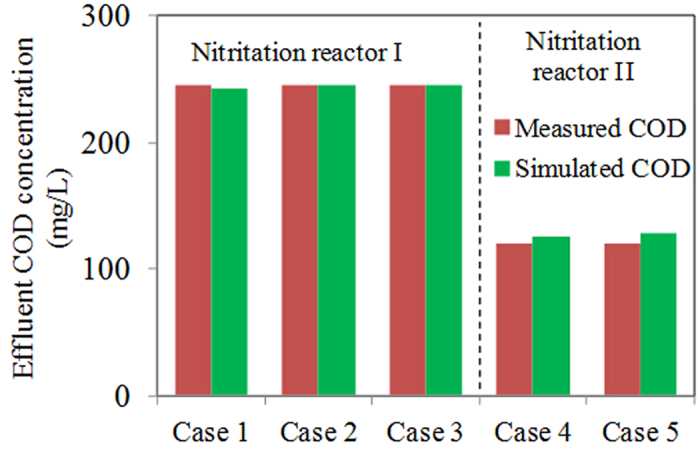
Measured and simulated effluent COD data in Nitritation reactors I and II. Case 1: DO = 0.5 mg O_2_/L in Nitritation reactor I; Case 2: DO = 1.0 mg O_2_/L in Nitritation reactor I; Case 3: DO = 3.0 mg O_2_/L in Nitritation reactor I; Case 4; Data on 16 March 2011 in Nitritation reactor II; Case 5: Data on 21 March 2011 in Nitritation reactor II.

A two-step procedure was applied to calibrate the model. In the first step, the kinetic parameters related to AOB were tested using the ammonium and nitrite data. Then, the maximum COD oxidation rate (r_COD,max_), was further calibrated using the volumetric N_2_O emission rate and effluent COD data in the second step. The calibration procedure is shown in Fig. S1. In this work, the default kinetic parameters related to AOB could describe the nitrogen conversion profiles well (Fig. 1A). Therefore, there is no need to calibrate the kinetic parameters associated with AOB. We then calibrated r_COD,max_. The calibration of the r_COD,max_ involved optimizing the value of this parameter by fitting the simulation results to the experiment data from Nitritation reactor I at DO = 0.5 mg O_2_/L. The measured and simulated N_2_O dynamics, along with the effluent COD data are illustrated in Figs 1 and 2. The model captured all these trends well. The good agreement between these simulated and measured NH_4_^+^, NO_2_^−^, effluent COD and N_2_O dynamics supported that the model could be used to estimate N_2_O production from the nitritation reactors receiving anaerobic digestion liquor.

The calibrated value of r_COD,max_, which gives the optimum model fittings with the experimental data, is listed in Table S1, together with its 95% confidence interval. The calibrated r_COD,max_ value of 1.33 ± 0.02 mmol COD/(gVSS^*^h) (with 95% confidence interval) (VSS: volatile suspended solids) is much smaller than that obtained (8.46 mmol COD/(gVSS*h)) by Pan *et al.*^20^. This is likely because only 35% of the biomass were HB in this study^3^, whereas the majority (>90%) of the biomass were HB in the study of Pan *et al.*^20^. Also, the anaerobic digestion liquor and the product of biomass decay were the source of COD in this study, which is difficult to be utilized^23^. In contrast, the readily biodegradable methanol was used as COD source in the study of Pan *et al.*^20^. The 95% confidence interval of r_COD,max_ (0.02 mmol COD/(gVSS*h)) is approximately 2% of the estimated value (1.33 mmol COD/(gVSS*h)), indicating that parameter r_COD,max_ has a high-level of identifiability and the estimated value is reliable.

### Model validation and further evaluation

**Figure 3:**
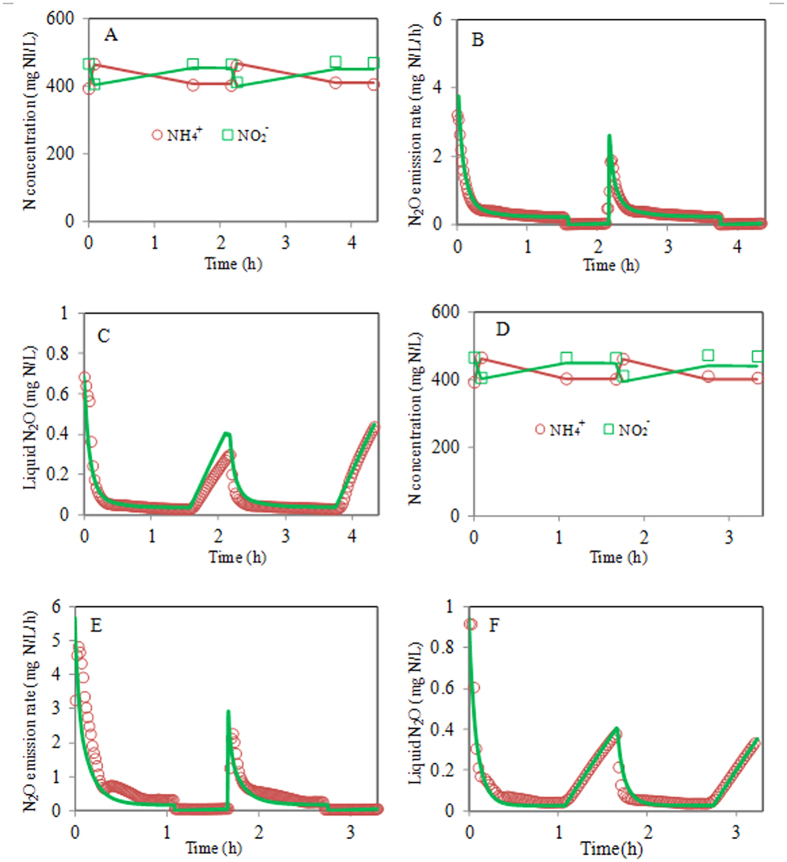
Model validation results using the experimental data from Nitritation reactor I at DO = 1.0 mg O_2_/L (**A**–**C**) and 3.0 mg O_2_/L (**D**–**F**) (measured data: symbols; model predictions: lines). (**A**,**D**) NH_4_^+^ and NO_2_^−^ data; (**B**,**E**) Volumetric N_2_O emission rate; (**C**,**F**) Liquid phase N_2_O concentration.

Model validation was based on the comparison between the model predictions (using the same value of r_COD,max_ as obtained in model calibration) and the experimental data from Nitritation reactor I under different DO conditions (i.e. 1.0 and 3.0 mg O_2_/L). The model predictions and the experimental results are shown in Figs 2 and 3. The validation results show that the model predictions match the measured effluent COD, NH_4_^+^, NO_2_^−^ and N_2_O dynamics well in all these validation experiments, which supports the validity of the N_2_O model.

**Figure 4:**
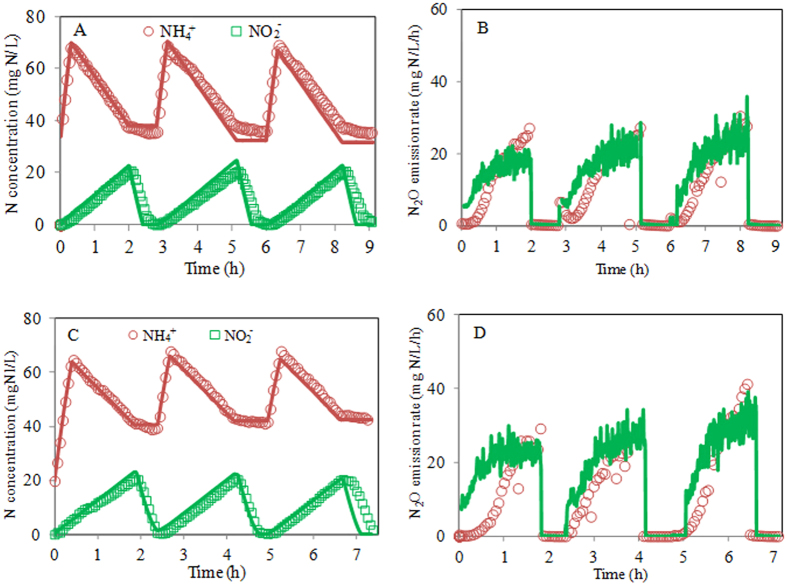
Model calibration and validation results using the experimental data from Nitritation reactor II (measured data: symbols; model predictions: lines). (**A**,**B**) NH_4_^+^, NO_2_^−^ and volumetric N_2_O emission rate results for calibration; (**C**,**D**) NH_4_^+^, NO_2_^−^ and volumetric N_2_O emission rate results for validation.

To further evaluate the N_2_O model, the experimental results of the effluent COD, NH_4_^+^, NO_2_^−^ and N_2_O dynamics from Nitritation reactor II were also used. It should be noted that Nitritation reactor I was conducting partial nitritation, whereas Nitritation reactor II was performing nitritation-denitritation. They represented two different treatment options of the anaerobic digestion liquor. The experimental results collected on 16 March 2011 were used to recalibrate r_COD,max_, and the results collected on 21 March 2011 were used for model validation. The value of r_COD,max_ was first recalibrated. The obtained value of r_COD,max_ for Nitritation reactor II is 4.78 ± 1.35 mmol COD/(gVSS^*^h) (with 95% confidence interval). This value is higher than that (1.33 ± 0.02 mmol COD/(gVSS*h)) obtained for Nitritation reactor I. This indicates that the COD in the anaerobic digestion liquor of Nitritation reactor II is easier to be biodegraded than that of Nitritation reactor I. The validation of the resulting value of r_COD,max_ was further performed through comparison of the model predictions with the experimental data of NH_4_^+^, NO_2_^−^, effluent COD and N_2_O dynamics collected on 21 March 2011. Figure 4 shows the modeling and experimental results of the model calibration (Fig. 4A,B) and model validation (Fig. 4C,D). As can be seen in Fig. 3, the model predictions are consistent with the experimental results and no systematic deviations are observed, further suggesting that the model is appropriate for describing the N_2_O production in the nitritation reactor fed with anaerobic digestion liquor.

## Discussion

In this work, a mathematical model including both AOB and HB is constructed to predict N_2_O production from the nitritation reactors receiving real anaerobic digestion liquor. This is for the first time that N_2_O production from such systems was modeled considering both AOB and HB. The kinetic parameter (r_COD,max_) closely related to N_2_O production by HB was estimated from the experimental data. The value obtained was robust in its ability to predict N_2_O dynamics. The validity of this model was confirmed by the independent NH_4_^+^, NO_2_^−^, COD and N_2_O data from both the lab-scale and the pilot-scale nitritation reactors receiving real anaerobic digestion liquor. The successful application of the model in this work indicates that it is applicable to describe N_2_O production in the nitritation reactors receiving anaerobic digestion liquor.

We also performed additional simulation studies using only the AOB N_2_O model^9^ to evaluate the measured N_2_O data from the two nitritation reactors used in this work. The model parameters for the AOB N_2_O model were the same as those in Nitritation reactors I and II. The results showed that this model could not reproduce the measured N_2_O data (see Fig. S2). This is due to the fact that both AOB and HB play a role in N_2_O production in the nitritation systems receiving real anaerobic digestion liquor. This is in contrast to the conclusion of other studies^8^,^12^,^14^, in which the contribution of HB to N_2_O production was assumed to be negligible.

**Figure 5:**
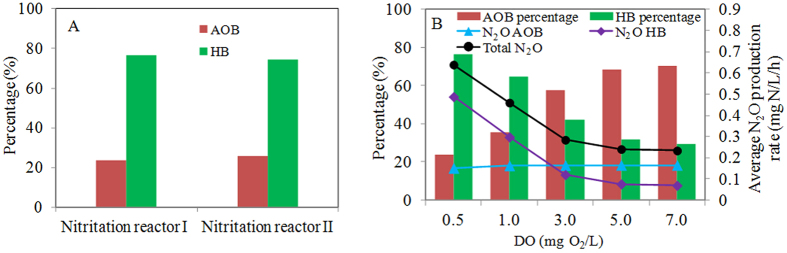
Model predictions of (**A**) the contributions of AOB and HB to N_2_O production in Nitritation reactors I and II, and (**B**) average aerobic N_2_O production rates in Nitritation reactors I at various DO levels.

Figure 5A shows the model predicted contributions of AOB and HB to the N_2_O production from Nitritation reactors I and II. HB are the dominant contributors to the N_2_O production, accounting for approximately 75% in both Nitritation reactors I (DO = 0.5 mg O_2_/L) and II (DO = 0.5–1.0 mg O_2_/L). In contrast, only 25% of N_2_O production can be attributed to AOB. This for the first time quantifies the contribution of HB and AOB to the N_2_O production in the nitritation systems receiving real anaerobic digestion liquor.

The contributions of AOB and HB to the aerobic N_2_O production at different DO concentrations are also evaluated using Nitritation reactor I as an example. Figure 5B shows that the contribution of HB to the aerobic N_2_O production decreases from 75% at DO = 0.5 mg O_2_/L to 25% at DO = 7.0 mg O_2_/L, with a corresponding increase of AOB contribution (from 25% to 75%). These results suggest that AOB are the dominant contributor to aerobic N_2_O production only when DO in the nitritation systems is high (e.g. > 3.0 mg O_2_/L), whereas HB would be responsible for the majority of the N_2_O production at low DO levels. Wang *et al.*^3^ indicated that both AOB and HB contributed to the N_2_O production in Nitritation reactor I and the contribution of HB to the N_2_O production decreased with increasing DO. Our modeling results support the observations made in Wang *et al.*^3^. Figure 5B also shows the average aerobic N_2_O production rates of both AOB and HB at various DO levels. In general, aerobic N_2_O production rate of AOB increases with increasing DO, which is in agreement with results reported by Law *et al.*^24^. In contrast, aerobic N_2_O production rate of HB decreases with increasing DO, from 0.49 mg N/L/h at DO = 0.5 mg O_2_/L to 0.07 mg N/L/h at DO = 7.0 mg O_2_/L. This is due to the fact that higher DO inhibits heterotrophic nitrite reduction, thereby decreasing N_2_O production by HB. Figure 5B also shows that the total N_2_O production rate (i.e. N_2_O production rate of both AOB and HB) decreased dramatically from 0.65 to 0.28 mg N/L/h when DO concentration increased from 0.5 to 3.0 mg O_2_/L. Afterwards, the total N_2_O production rate remained relatively stable at approximately 0.25 mg N/L/h even if DO concentration kept increasing from 3.0 to 7.0 mg O_2_/L. In contrast, the ammonium oxidizing rate kept increasing when DO concentration increased from 0.5 to 3.0 mg O_2_/L, but remained stable when DO concentration increased from 3.0 to 7.0 mg O_2_/L (see Fig. S3). This indicates that DO should be maintained at around 3.0 mg O_2_/L from the perspective of minimizing N_2_O production rate and maximizing ammonium oxidizing rate. However, while increasing DO to decrease N_2_O production rate and increase ammonium oxidizing rate, energy consumption would increase accordingly. This will increase operating costs. Therefore, a trade-off has to be made in practice between minimizing N_2_O production rate and minimizing operating costs.

It should be noted that the potential existence of NOB was not considered in the current model. This is acceptable due to the fact that only a tiny amount of nitrate was produced (less than 10% of ammonium oxidized) in the studied systems. Also, NOB are known not to contribute to N_2_O production^7^. However, the processes related to NOB could be easily incorporated into the model based on the study of Moussa *et al.*^25^ if necessary in future applications. It should also be pointed out that the biomass growth of AOB and HB was negligible in a short batch test of a few hours. Therefore, the simulation results regarding their concentrations were not shown in the study. Also, only r_COD,max_ regarding N_2_O production by HB was estimated from the experimental data. The parameters with regard to AOB (e.g. 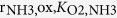) were not calibrated in this work because the adopted values from literature were able to describe the NH_4_^+^ and NO_2_^−^ data well. These parameter values could be system-specific, and may need calibration when the model is applied to other systems. It should be highlighted that a free nitrous acid (FNA i.e. HNO_2_)-related Haldane-type kinetics was added to the model structure (see Section of “Mathematical model for N_2_O production) to describe the heterotrophic N_2_O reduction. This is necessary for modeling heterotrophic N_2_O reduction in the nitritation systems receiving anaerobic digestion liquor. FNA has been reported to have an inhibitory effect on heterotrophic N_2_O reduction^26^. For instance, Zhou *et al.*^26^ demonstrated that FNA inhibition on N_2_O reduction initiated at an FNA concentration of 0.0002 mg HNO_2_-N/L. When the FNA concentration was greater than 0.004 mg HNO_2_-N/L, N_2_O reduction could be completely inhibited by FNA. The FNA concentrations in the Nitritation reactors I and II could reach 0.32 and 0.009 mg HNO_2_-N/L, respectively (see Fig. S4 for the FNA concentrations in Nitritation reactors I and II). Therefore, FNA inhibition would occur.

In conclusion, a mathematical model including both ammonium-oxidizing bacteria (AOB) and heterotrophic bacteria (HB) is constructed to predict N_2_O production from the nitritation reactors receiving real anaerobic digestion liquor for the first time. Model calibration and validation show good agreement between the simulation results and the experimental results obtained from both lab- and pilot-scale nitritation reactors receiving real digestion liquor. HB are the dominant contributors to the N_2_O production in both reactors. The contribution of HB to aerobic N_2_O production decreases with increasing DO levels, with a corresponding increase of AOB contribution. Also, the N_2_O production rate of HB decreased with the increasing DO levels, whereas the N_2_O production rate of AOB increased when DO concentration increased. The model is expected to enhance our ability to predict N_2_O production from such systems.

## Materials and methods

### Experimental data for model evaluation

#### Nitritation reactor I

Experimental data from a culture performing partial nitritation previously reported in Wang *et al.*^3^ were used for the model calibration and validation. The culture was developed in a 4-L lab-scale sequencing batch reactor (SBR) fed with anaerobic digestion liquor collected from an Australian wastewater treatment plant. The anaerobic digestion liquor contained approximately 860 ± 13 mg NH_4_^+^-N/L and 345 ± 15 mg COD/L with the other composition described in Wang *et al.*^3^. The SBR was operated with a cycle time of 6 h, consisting of 5 min aerobic feeding I, 120 min aerobic reaction I, 35 min anoxic reaction I, 5 min aerobic feeding II, 120 min aerobic reaction II, 35 min anoxic reaction II, 2 min aerobic sludge wasting, 25 min settling, 8 min decanting and 5 min anoxic mixing. 0.5 L of anaerobic digestion liquor was fed in each feeding period giving a hydraulic retention time (HRT) of 24 h. The sludge retention time (SRT) was kept at 11 days. The temperature was controlled at 33 ± 1 °C using a water jacket. During the feeding, aerobic reaction and wasting phases, aeration was supplied to maintain a DO concentration of around 0.5 mg O_2_/L using an on/off controller. The pH in the SBR varied between 6.4 and 7.1 during a typical cycle with NaHCO_3_ solution (1 M) being added (0.1–3.0 ml per cycle) automatically via a programmable logic controller (PLC) when pH was below 6.4. This culture converted approximately 50 ± 5% of the NH_4_^+^-N to NO_2_^−^-N, resulting in both effluent ammonium and nitrite concentrations of 430 ± 40 mg N/L. Effluent nitrate was below 10 mg N/L at all times in the SBR. The effluent COD was 245 ± 16 mg/L. Microbial community analysis revealed that AOB accounted for 65% of the entire microbial community, with the other 35% being HB. In contrast, NOB were not detected (<1%). Mixed liquor suspended solids (MLSS) and mixed liquor volatile suspended solids (MLVSS) concentrations were 750 ± 30 mg/L and 610 ± 30 mg/L, respectively. Both the gas and liquid phase N_2_O were monitored every 3–4 days using an on-line gas analyzer and a liquid microsensor, respectively. More details of the reactor operation and performance can be found in Wang *et al.*^3^.

In addition, two tests were conducted to evaluate the effect of DO concentrations on aerobic N_2_O production. In these tests, DO concentrations in the aerobic phases of the SBR were controlled at around 1.0 and 3.0 mg O_2_/L, respectively, by adjusting air flow rate (2 L/min) and using an on/off controller. As the bacterial activity increases with the increasing DO concentrations, length of the aerobic phase was shortened to make sure that the effluent ammonium, and nitrite concentrations were comparable with those achieved at the DO level of 0.5 mg O_2_/L. Other operating conditions were the same as those at DO = 0.5 mg O_2_/L. More details can be found in Wang *et al.*^3^.

The gas phase N_2_O concentration was analyzed with an infrared analyzer (URAS 14 Advance Optima, ABB). The data were logged every 3 s. The liquid phase N_2_O was measured online using a N_2_O microsensor (N_2_O-100, Unisense A/S. Aarhus, Denmark). The N_2_O emission rate (mg N_2_O-N/h) was calculated by multiplying the gas phase N_2_O concentration by the known gas flow rate. The volumetric N_2_O emission rate (mg N_2_O-N/L/h) was calculated by dividing the N_2_O emission rate by the volume of the mixed liquor in the SBR. Mixed liquor samples were taken periodically using a syringe and immediately filtered through disposable Millipore filters (0.22 μm pore size) for NH_4_^+^, NO_2_^−^ and NO_3_^−^ analyses using a Lachat QuikChem8000 Flow Injection Analyzer. COD was analyzed according to Standard Methods^27^.

#### Nitritation reactor II

Experimental data from a culture performing nitritation-denitritation previously reported in Lemaire *et al.*^28^ were used to further evaluate the model. The culture was developed in a 500-L pilot-scale SBR receiving anaerobic digestion liquor from a French WWTP. The anaerobic digestion liquor contained approximately 475 ± 40 mg NH_4_^+^-N/L and 180 ± 50 mg COD/L with the other composition described in Lemaire *et al.*^28^. The SBR cycle was divided into several sub-cycles with each composed of aerobic feeding, aerobic reaction and anoxic reaction. Ethanol was added as a carbon source at the beginning of each anoxic reaction to provide enough carbon sources for nitrogen removal. After reaching the working volume of the SBR, the sub-cycle was completed. This was followed by a short sludge wastage phase to keep the SRT at around 15–20 days, a 1 h settling phase and a decanting phase to discard the treated effluent. The SBR was operated at 20–25 °C. During the aerobic feeding and aerobic reaction phases, DO was between 0.5 and 1.0 mg O_2_/L. pH varied between 6.7 and 7.1 during the cycle. Ammonium, nitrite and nitrate were measured by on-line sensors. This culture achieved total nitrogen removal of 85–90% and the effluent COD was around 120 mg/L. The gas phase N_2_O was measured continuously using an infrared analyzer (VA-3000, Horiba, Japan). More details of the reactor operation and performance can be found in Lemaire *et al.*^28^. The calculation of the N_2_O emission rate was similar to that for Nitritation reactor I.

### Mathematical model for N_2_O production

The previously proposed EC-based N_2_O models by Ni *et al.*^9^ and Pan *et al.*^20^ were integrated for the first time to describe the N_2_O data. In the N_2_O model developed by Ni *et al.*^9^, the NH_2_OH oxidation and AOB denitrification pathways were integrated to describe N_2_O production by AOB. In this model, the oxidation and reduction processes were modeled separately, with intracellular electron carriers introduced to link the two types of processes (see Table S1–S4). In the N_2_O model developed by Pan *et al.*^20^, the electron competition by different denitrification steps was described for the first time, through modeling the carbon oxidation and nitrogen reduction processes separately (see Table S1–S4). Electron carriers were introduced to link carbon oxidation and nitrogen oxides reduction. In addition, the biomass decay process was added to describe the generation of slowly biodegradable COD after cell death. The generated slowly biodegradable COD can then be hydrolyzed to readily biodegradable COD, which can be further utilized to produce the reduced form of the electron carrier during COD oxidation by HB. As free nitrous acid (FNA i.e. HNO_2_) has an inhibitory effect on N_2_O reduction^26^ (see Fig. S4 for FNA concentrations in Nitritation reactors I and II), a Haldane-type kinetics (S_HNO2_/(K_HNO2_ + S_HNO2_ + (S_HNO2_)^2^/K_I,HNO2_)) was applied to describe the N_2_O reduction (R14 in Table S3). Because the nitrite concentration in Nitritation reactor I was between 390 and 500 mg N/L and a high level of nitrite (>50 mg N/L) has an inhibitory effect on N_2_O production^29^, a Haldane-type kinetics (S_NO2_^−^/(K_NO2_^−^ + S_NO2_^−^ + (S_NO2_^−^)^2^/K_I,NO2_^−^)) was added to describe the NO_2_^−^ reduction for N_2_O production in Nitritation reactor I (R6 in Table S3). Since ethanol is much more easily biodegradable than the other biodegradable COD that exists in the anaerobic digestion liquor and which is produced from biomass decay, the anoxic ethanol oxidation process 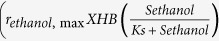 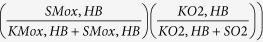 (see Tables S2 and S4) was introduced to model anoxic ethanol oxidation in Nitritation reactor II. The components, kinetic rate expressions, stoichiometric matrix, and kinetic and stoichiometric parameters of the N_2_O model are summarized in Table S1–S4.

The biomass concentrations of AOB and HB for model input were calculated based on microbial community analysis results and MLVSS concentration. The measured MLVSS concentration was apportioned to the bacterial populations based on the determined fractions of various bacterial populations including AOB and HB. This is the commonly used method of determining the concentrations of bacterial populations^9^,^30^. The biomass concentrations of AOB and HB were then determined as 210 mg/L and 400 mg/L, respectively, in Nitritation reactor I. In Nitritation reactor II, the biomass concentrations of AOB and HB were approximately 280 mg/L and 1600 mg/L, respectively.

### Model calibration and validation

The N_2_O model includes 45 stoichiometric and kinetic parameters, as summarized in Table S1. Most of these parameters were well established in previous studies. Therefore, literature values were directly adopted for these parameters (Table S1). As the N_2_O production pathway by HB is for the first time included in the model for estimating N_2_O production from nitritation systems receiving anaerobic digestion liquor and sensitivity analysis reveals that the maximum COD oxidation rate (r_COD,max_) is the key parameter governing N_2_O production by HB, r_COD,max_ was estimated using the experimentally obtained volumetric N_2_O emission rate and COD data.

Experimental data from Nitritation reactor I at DO = 0.5 mg O_2_/L were used to calibrate the model. The value of r_COD,max_ was estimated by minimizing the sum of squares of the deviations between the measured data and the model predictions using a modified version of AQUASIM 2.1d^31^, with 95% confidence interval for parameter uncertainty analysis. The 95% confidence interval of r_COD,max_ was calculated from the mean square fitting error. Model validation was then carried out with the calibrated value of r_COD,max_ using the other two sets of experimental data under different DO conditions (i.e. 1.0 and 3.0 mg O_2_/L).

To further verify the validity and applicability of the N_2_O model, we also applied the model to evaluate the N_2_O data from Nitritation reactor II. The r_COD,max_ was recalibrated using one cycle data monitored on 16 March 2010 from Nitritation reactor II. The validation step was then carried out with the recalibrated r_COD,max_ using one cycle monitoring data on 21 March 2010 from Nitritation reactor II with dynamic influent conditions which has not been used to estimate the parameter.

## Additional Information

**How to cite this article**: Wang, Q. *et al.* Modeling of Nitrous Oxide Production from Nitritation Reactors Treating Real Anaerobic Digestion Liquor. *Sci. Rep.* **6**, 25336; doi: 10.1038/srep25336 (2016).

## Supplementary Material

Supplementary Information
