# Supplementary material for: Modeling of Nitrous Oxide Production from Nitritation Reactors Treating Real Anaerobic Digestion Liquor
Source: Sci Rep. 2016 Apr 29;6:25336. doi: 10.1038/srep25336 (PMC4850461; doi:10.1038/srep25336)
Supplement: Supplementary Information [file srep25336-s1.pdf]

Modeling of Nitrous Oxide Production from Nitritation Reactors Treating Real Anaerobic Digestion Liquor

(Supplementary Information)

Qilin Wang[1], Bing-Jie Ni[1], Romain Lemaire[2], Xiaodi Hao[3] and Zhiguo Yuan[1]*

[1]Advanced Water Management Centre (AWMC), The University of Queensland, QLD 4072, Australia

[2] Veolia Technical and Performance Department, St-Maurice, France

[3]Key Laboratory of Urban Stormwater System and Water Environment/R&D Centre for Sustainable Wastewater Treatment (Beijing University of Civil Engineering and Architecture), Ministry of Education, Beijing 100044, P.R. China

Correspondence and requests for materials should be addressed to Z.Y. (email: zhiguo@awmc.uq.edu.au)

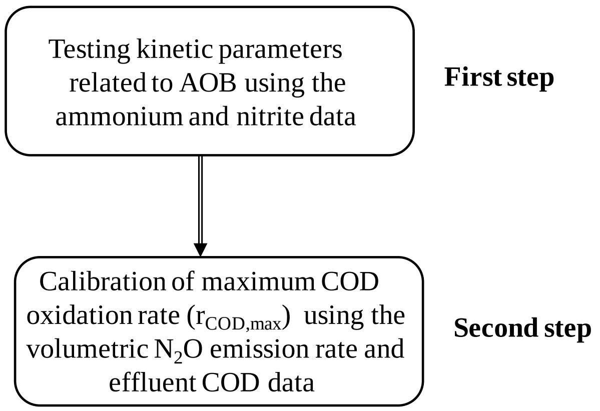

Figure S1. Two-step calibration procedure applied in this study.

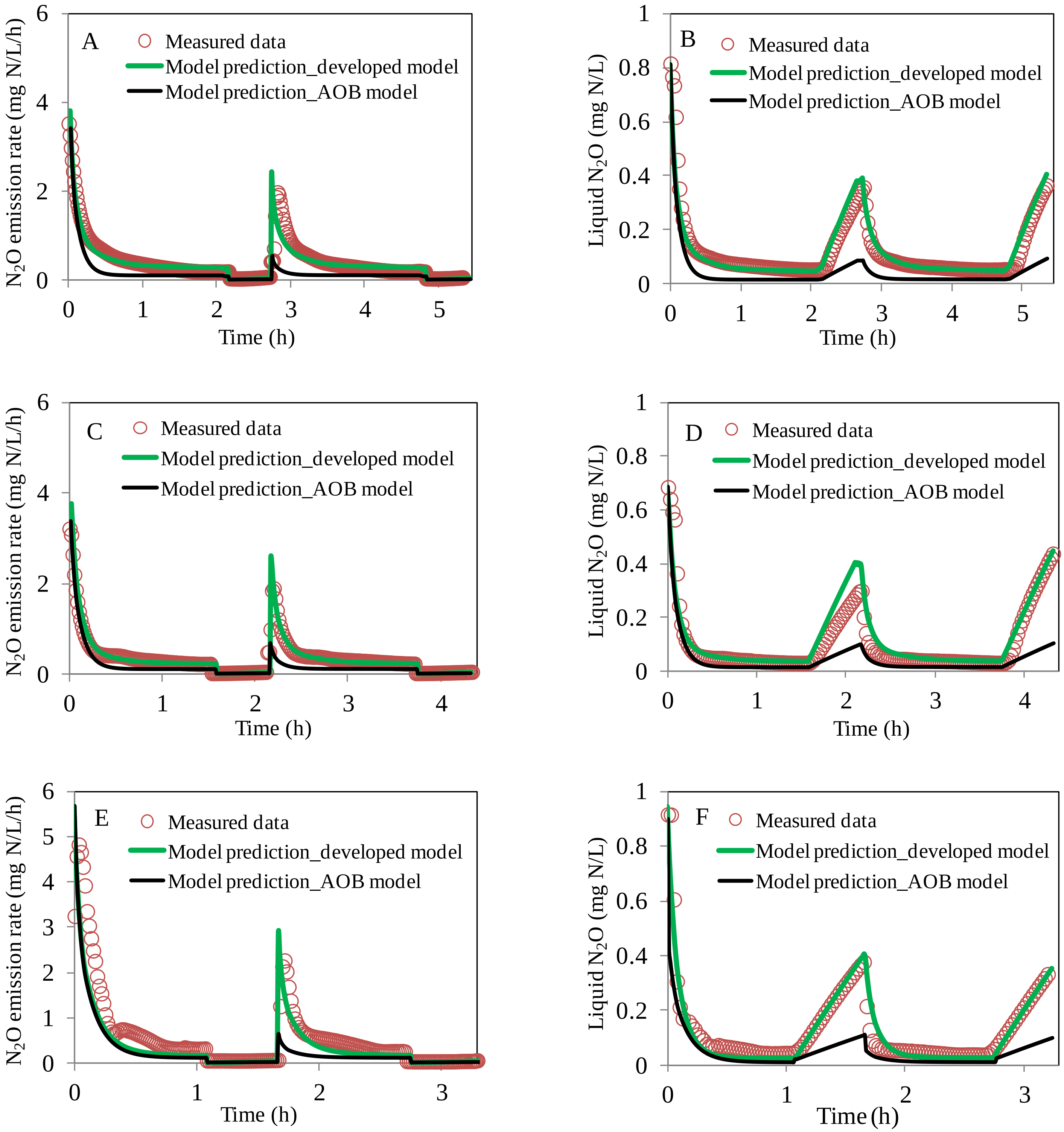
A
Measured data
Model prediction_developed model
Model prediction_AOB model
N2O emission rate (mg N/L/h)
Time (h)
B
Measured data
Model prediction_developed model
Model prediction_AOB model
Liquid N2O (mg N/L)
Time (h)
C
Measured data
Model prediction_developed model
Model prediction_AOB model
N2O emission rate (mg N/L/h)
Time (h)
D
Measured data
Model prediction_developed model
Model prediction_AOB model
Liquid N2O (mg N/L)
Time (h)
E
Measured data
Model prediction_developed model
Model prediction_AOB model
N2O emission rate (mg N/L/h)
Time (h)
F
Measured data
Model prediction_developed model
Model prediction_AOB model
Liquid N2O (mg N/L)
Time (h)

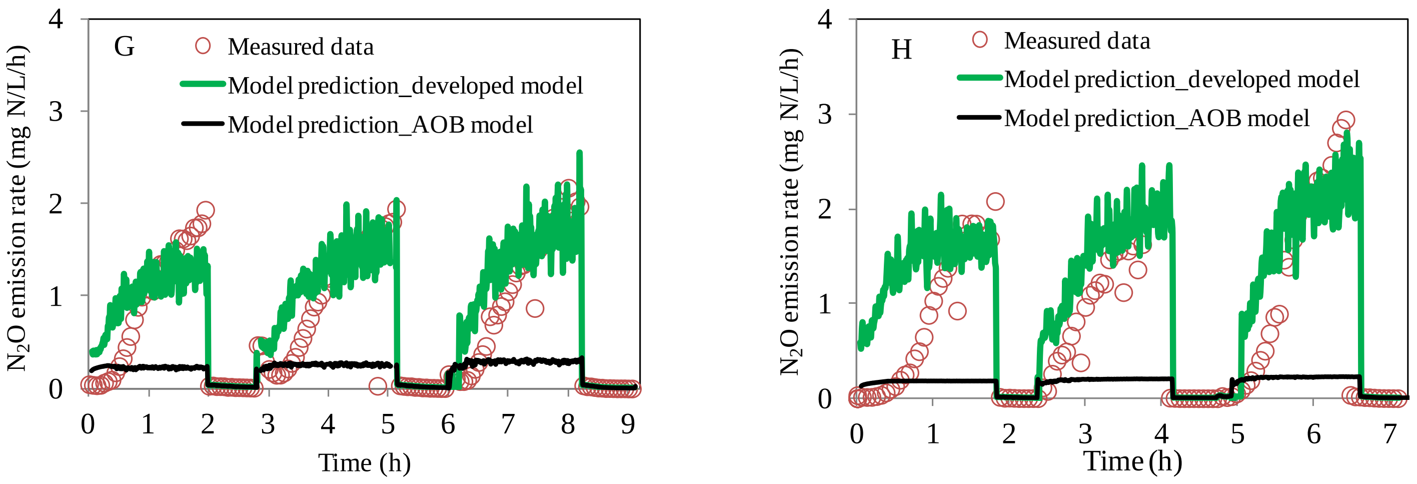

Figure S2. Comparison of the developed model with the AOB model[1] in terms of N_2O emission rate for Nitritation reactor I at DO=0.5 mg O_2/L (A, B), DO=1.0 mg O_2/L (C, D) and DO=3.0 mg O_2/L (E, F), and Nitritation reactor II on 16 March 2010 (G) and 21 March 2010 (H).

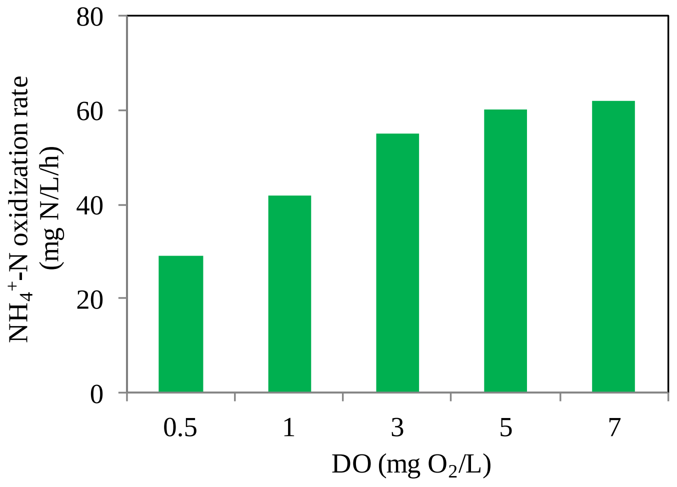

Figure S3. Ammonium oxidization rate in Nitritation reactor I at various DO concentrations.

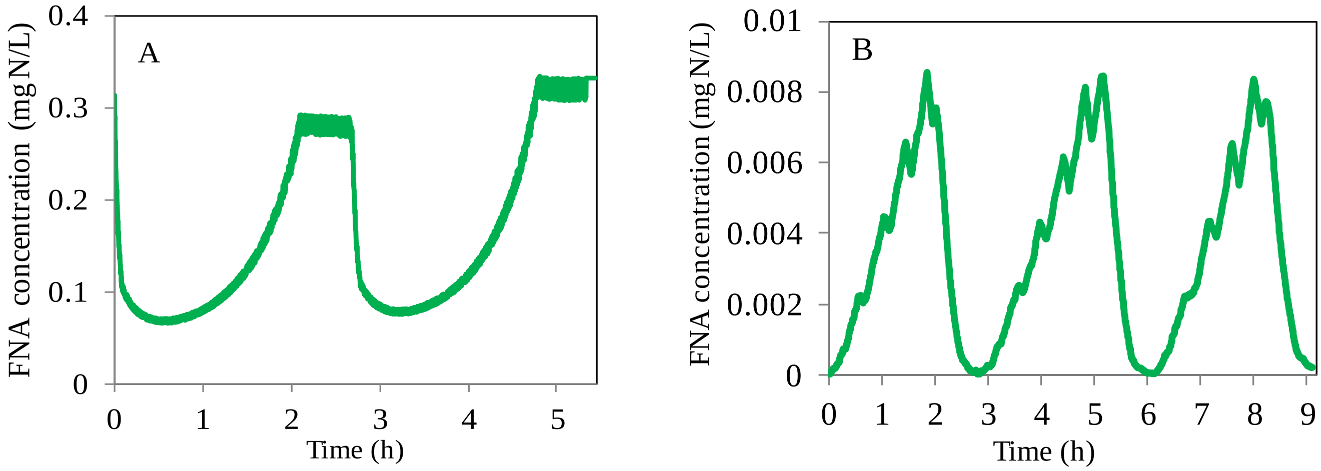

Figure S4. FNA concentration in Nitritation reactors I and II. (A) Nitritation reactor I at DO=0.5 mg/L; (B) Nitritation reactor II on 16 March 2010.

Table S1. Kinetic and stoichiometric parameters of the N_2O model

Parameter	Definition	Value	Source
$r_{NH3,ox}$	Maximum ammonia oxidation rate (mmol N/(gVSS*h)	14.75	Ni et al.[1]
$r_{NH2OH,ox}$	Maximum NH_2OH oxidation rate (mmol N/(gVSS*h))	22.86	Ni et al.[1]
$r_{NO,ox}$	Maximum NO oxidation rate (mmol N/(gVSS*h))	22.86	Ni et al.[1]
$r_{O2,red}$	Maximum oxygen reduction rate (mmol O_2/(gVSS*h)	48.02	Ni et al.[1]
$r_{NO2,red}$	Maximum nitrite reduction rate (mmol N/(gVSS*h))	3.06	Ni et al.[1]
$r_{NO,red}$	Maximum NO reduction rate (mmol N/(gVSS*h))	0.016	Ni et al.[1]
$K_{O2,NH3}$	Oxygen affinity constant for ammonia oxidation (mmol O_2/L)	0.019	Ni et al.[1]
K_{NH3}	Ammonia affinity constant for ammonia oxidation (mmol N /L)	0.17	Ni et al.[1]
K_{NH2OH}	NH_2OH affinity constant for NH_2OH oxidation (mmol N/L)	0.05	Ni et al.[1]
$K_{NO,ox}$	NO affinity constant for NO oxidation (mmol N/L)	0.0006	Ni et al.[1]
$K_{O2,red}$	Oxygen affinity constant for oxygen reduction (mmol O_2/L)	0.0019	Ni et al.[1]
$K_{NO2,AOB}$	Nitrite affinity constant for nitrite reduction by AOB (mmol N/L)	0.01	Ni et al.[1]
$K_{NO,red}$	NO affinity constant for NO reduction by AOB (mmol N/L)	0.0006	Ni et al.[1]
$K_{Mox,AOB}$	Affinity constant for S_{Mox}, R3 (mmol/(g VSS))	0.0001	Ni et al.[1]
$K_{Mred,1,AOB}$	Affinity constant for S_{Mred}, R1 (mmol/(g VSS))	0.00001	Ni et al.[1]
$K_{Mred,2,AOB}$	Affinity constant for S_{Mred}, R4 (mmol/(g VSS))	0.00001	Ni et al.[1]
$K_{Mred,3,AOB}$	Affinity constant for S_{Mred}, R5 (mmol/(g VSS))	0.069	Ni et al.[1]
$K_{Mred,4,AOB}$	Affinity constant for S_{Mred}, R6 (mmol/(g VSS))	0.19	Ni et al.[1]
$K_{I,NO2}$	Nitrite inhibition constant for nitrite reduction (mmol N/L)	13	Ni et al.[1]
b_{AOB}	Decay rate of AOB (h^{-1})	0.00625	Henze et al.[2]
$C_{tot,AOB}$	Total electron carrier concentration of AOB (mmol/g VSS)	0.01	Ni et al.[1]
$r_{COD,max}$	Maximum COD oxidation rate (mmol COD/(gVSS*h)	1.33 ± 0.02[a]/ 4.78 ± 1.35[b]	Estimated
$r_{ethanol,max}$[c]	Maximum ethanol oxidation rate (mmol COD/(gVSS*h)	8.46	Pan et al.[3]
$r_{NO3,max}$	Maximum nitrate reduction rate (mmol N /(gVSS*h))	3.99	Pan et al.[3]
$r_{NO2,max}$	Maximum nitrite reduction rate (mmol N /(gVSS*h))	5.27	Pan et al.[3]
$r_{NO,max}$	Maximum NO reaction rate (mmol N/(gVSS*h))	50	Pan et al.[3]
$r_{N2O,max}$	Maximum N_2O reaction rate (mmol N/(gVSS*h))	20	Pan et al.[3]
k_H	Hydrolysis rate constant (mmol COD /(gVSS*h))	3.9	Henze et al.[2]
K_X	Hydrolysis saturation constant (mmol COD/g VSS)	3.125	Henze et al.[2]
K_S	Affinity constant for Ss (mmol COD/L)	0.625	Pan et al.[3]
$K_{NO3,HB}$	Affinity constant for nitrate (mmol N/L)	0.018	Pan et al.[3]
$K_{NO2,HB}$	Affinity constant for nitrite (mmol N/L)	0.0041	Pan et al.[3]
$K_{NO,HB}$	Affinity constant for NO (mmol N/L)	0.000011	Pan et al.[3]
$K_{N2O,HB}$	Affinity constant for N_2O (mmol N/L)	0.0025	Pan et al.[3]
$K_{Mox,HB}$	Affinity constant for S_{Mox}, R9 (mmol/(g VSS))	0.0001	Pan et al.[3]
$K_{Mred,1,HB}$	Affinity constant for S_{Mred}, R11 (mmol/(g VSS))	0.00458	Pan et al.[3]
$K_{Mred,2,HB}$	Affinity constant for S_{Mred}, R12 (mmol/(g VSS))	0.000393	Pan et al.[3]
$K_{Mred,3,HB}$	Affinity constant for S_{Mred}, R13 (mmol/(g VSS))	0.00001	Pan et al.[3]
$K_{Mred,4,HB}$	Affinity constant for S_{Mred}, R14 (mmol/(g VSS))	0.00323	Pan et al.[3]
K_{HNO2}	Affinity constant for HNO_2 (mmol N/L)	0.0000022	Zhou et al.[4]
$K_{I,HNO2}$	HNO_2 inhibition constant for N_2O reduction (mmol N/L)	0.000136	Zhou et al.[4]
$K_{O2,HB}$	Half-saturation coefficient for oxygen (mmol O_2/L)	0.003125	Hiatt and Grady,[5]
η	Reduction factor for denitrification	0.8	Henze et al.[2]
b_H	Decay rate of HB (h^{-1})	0.00833	Henze et al.[2]
Y_H	Heterotrophic yield (g COD/g COD)	0.625	Henze et al.[2]
$C_{tot,HB}$	Total electron carrier concentration of HB (mmol/(g VSS))	0.01	Pan et al.[3]

[a] Value in Nitritation reactor I
[b] Value in Nitritation reactor II
[c] $r_{ethanol,max}$ was only used in Nitritation reactor II

Table S2. Definition and units of the N_2O model components

Variable	Definition	Unit
S_{O2}	Dissolved oxygen	mmol O_2/L
S_{NH3}	Ammonia	mmol N/L
S_{NO2}	Nitrite	mmol N/L
S_{NO}	NO	mmol N/L
S_{N2O}	N_2O	mmol N/L
S_{NH2OH}	NH_2OH	mmol N/L
$S_{Mred,AOB}$	Reduced form of electron carrier in AOB	mmol/L
$S_{Mox,AOB}$	Oxidized form of electron carrier in AOB	mmol/L
X_{AOB}	Active AOB	g VSS/L
S_S	Readily biodegradable COD	mmol COD/L
X_S	Slowly biodegradable COD	mmol COD/L
S_{HNO2}	HNO_2 concentration	mmol N/L
$S_{Mred,HB}$	Reduced form of electron carrier in HB	mmol/L
$S_{Mox,HB}$	Oxidized form of electron carrier in HB	mmol/L
X_{HB}	Active HB	g VSS/L

Table S3. Kinetic rate expressions for the N_2O production model

Process	Kinetic rate expressions
**Ammonium-oxidizing bacteria (AOB)**	
R1: NH_3 oxidation to NH_2OH	$r_{NH3,OX} X_{AOB}(\frac{S_{O2}}{K_{O2,NH3}+S_{O2}})(\frac{S_{NH3}}{K_{NH3}+S_{NH3}})(\frac{S_{Mred,AOB}}{K_{Mred,1,AOB}+S_{Mred,AOB}})$
R2: NH_2OH oxidation to NO	$r_{NH2OH,OX} X_{AOB}(\frac{S_{NH2OH}}{K_{NH2OH}+S_{NH2OH}})(\frac{S_{Mox,AOB}}{K_{Mox,AOB}+S_{Mox,AOB}})$
R3: NO oxidation to NO_2^-	$r_{NO,OX} X_{AOB}(\frac{S_{NO}}{K_{NO,OX}+S_{NO}})(\frac{S_{Mox,AOB}}{K_{Mox,AOB}+S_{Mox,AOB}})$
R4: NO reduction to N_2O	$r_{NO,red} X_{AOB}(\frac{S_{NO}}{K_{NO,red}+S_{NO}})(\frac{S_{Mred,AOB}}{K_{Mred,2,AOB}+S_{Mred,AOB}})$
R5: O_2 reduction to H_2O	$r_{O2,red} X_{AOB}(\frac{S_{O2}}{K_{O2,red}+S_{O2}})(\frac{S_{Mred,AOB}}{K_{Mred,3,AOB}+S_{Mred,AOB}})$
R6: NO_2^- reduction to N_2O	$r_{NO2,red} X_{AOB}(\frac{S_{NO2}}{K_{NO2,AOB}+S_{NO2}})(\frac{S_{Mred,AOB}}{K_{Mred,4,AOB}+S_{Mred,AOB}})$ [a]
R7: Decay of AOB	$b_{AOB}*X_{AOB}$
E1: Equation of electron carriers for AOB	$S_{Mred,AOB}+S_{Mox,AOB}=C_{tot,AOB}$
**Heterotrophic bacteria (HB)**	
R8: Hydrolysis of Xs	$k_{HB} X_{HB}(\frac{Xs/X_{HB}}{K_X+Xs/X_{HB}})$
R9: Aerobic COD oxidation	$r_{COD,\max} X_{HB}(\frac{S_S}{Ks+Ss})(\frac{S_{Mox,HB}}{K_{Mox,HB}+S_{Mox,HB}})(\frac{S_{O2}}{K_{O2,HB}+S_{O2}})$
R10: Anoxic COD oxidation[b]	$r_{COD,\max} \eta X_{HB}(\frac{S_S}{Ks+Ss})(\frac{S_{Mox,HB}}{K_{Mox,HB}+S_{Mox,HB}})(\frac{K_{O2,HB}}{K_{O2,HB}+S_{O2}})$

R11: NO_3^- reduction to NO_2^-	$r_{NO3,\max} X_{HB}(\frac{S_{NO3}}{K_{NO3,HB}+S_{NO3}})(\frac{S_{Mred,HB}}{K_{Mred,1,HB}+S_{Mred,HB}})(\frac{K_{O2,HB}}{K_{O2,HB}+S_{O2}})$
R12: NO_2^- reduction to NO	$r_{NO2,\max} X_{HB}(\frac{S_{NO2}}{K_{NO2,HB}+S_{NO2}})(\frac{S_{Mred,HB}}{K_{Mred,2,HB}+S_{Mred,HB}})(\frac{K_{O2,HB}}{K_{O2,HB}+S_{O2}})$
R13: NO reduction to N_2O	$r_{NO,\max} X_{HB}(\frac{S_{NO}}{K_{NO,HB}+S_{NO}})(\frac{S_{Mred,HB}}{K_{Mred,3,HB}+S_{Mred,HB}})(\frac{K_{O2,HB}}{K_{O2,HB}+S_{O2}})$
R14: N_2O reduction to N_2	$r_{N2O,\max} X_{HB}(\frac{S_{N2O}}{K_{N2O,HB}+S_{N2O}})(\frac{S_{Mred,HB}}{K_{Mred,4,HB}+S_{Mred,HB}})(\frac{K_{O2,HB}}{K_{O2,HB}+S_{O2}})(\frac{S_{HNO2}}{K_{HNO2}+S_{HNO2}+(S_{HNO2})^2/K_{I,HNO2}})$
R15: Decay of HB	$b_{HB}*X_{HB}$
E2: Equation of electron carriers for HB	$S_{Mred,HB}+S_{Mox,HB}=C_{tot,HB}$

[a] A Haldane-type inhibition term $(\frac{S_{NO2}}{K_{NO2,AOB}+S_{NO2}+(S_{NO2})^2/K_{I,NO2}})$ was applied to Nitritation reactor I since the nitrite concentration in Nitritation reactor I was between 390 and 500 mg N/L and a high level of nitrite (>50 mg N/L) has an inhibitory effect on N_2O production[6].

[b] Since ethanol is much more easily biodegradable than the other biodegradable COD that exists in the anaerobic digestion liquor and that produced from biomass decay, the anoxic ethanol oxidation process ($r_{ethanol,\max} X_{HB}(\frac{S_{ethanol}}{Ks+S_{ethanol}})(\frac{S_{Mox,HB}}{K_{Mox,HB}+S_{Mox,HB}})(\frac{K_{O2,HB}}{K_{O2,HB}+S_{O2}})$) ($r_{ethanol,max}$: Maximum ethanol oxidation rate, see Table S1) was introduced to model anoxic ethanol oxidation in Nitritation reactor II.

Table S4. Stoichiometric matrix for the integrated N_2O production model

Process	S_{O2}	S_{NH3}	S_{NH2OH}	S_{NO3}	S_{NO2}	S_{NO}	S_{N2O}	S_S	$S_{Mox,AOB}$	$S_{Mred,AOB}$	$S_{Mox,HB}$	$S_{Mred,HB}$	X_S	X_{AOB}	X_{HB}
	**Ammonium-oxidizing bacteria (AOB)**														
R1	-1	-1	1						1	-1					
R2			-1			1			-3/2	3/2					
R3					1	-1			-1/2	1/2					
R4						-1	1/2		1/2	-1/2					
R5	-1/2								1	-1					
R6					-1		1/2		1	-1					
R7													35.5	-1	
E1															
	**Heterotrophic bacteria (HB)**														
R8								1					-1		
R9								-1			$-(1-Y_H)$	$1-Y_H$			Y_H
R10								-1			$-(1-Y_H)$	$1-Y_H$			Y_H
R11				-1	1						1	-1			
R12					-1	1					1/2	-1/2			
R13						-1	1/2				1/2	-1/2			
R14							-1				1	-1			
R15													35.5		-1
E2															

References:

1. Ni, B.J., Peng, L., Law, Y., Guo, J. & Yuan Z. Modelling of nitrous oxide production by autotrophic ammonia-oxidizing bacteria with multiple production pathways. *Environ. Sci. Technol.* **48**, 3916-3924 (2014).

2. Henze, M., Gujer, W., Mino, T. & van Loosdrecht, M.C.M. 2000. Activated Sludge Models ASM1, ASM 2, ASM2d, and ASM 3. IWA Sc and Technical Report No. 9. IWA Publishing , London, UK.

3. Pan, Y., Ni, B.J. & Yuan, Z. Modeling electron competition among nitrogen oxides reduction and N_2O accumulation in denitrification. *Environ. Sci. Technol.* **47**, 11083-11091 (2013).

4. Zhou, Y., Pijuan, M., Zeng, R.J. & Yuan, Z. 2008. Free nitrous acid inhibition on nitrous oxide reduction by a denitrifying-enhanced biological phosphorus removal sludge. *Environ. Sci. Technol.* **42**, 8260−8265.

5. Hiatt, W.C. & Grady, C.P.L. An updated process model for carbon oxidation, nitrification, and denitrification. *Water Environ. Res.* **80**, 2145−2156 (2008).

6. Law, Y., Lant, P. A. & Yuan, Z. The confounding effect of nitrite on N_2O production by an enriched ammonia-oxidising culture. *Environ. Sci. Technol.* **47**, 7186 −7194 (2013).
